# Balancing benefits and challenges of eHealth for family caregivers of people with Parkinson’s disease – A qualitative study

**DOI:** 10.1177/20552076261447410

**Published:** 2026-04-29

**Authors:** Hanna Johansson, Anette Alvariza, Breiffni Leavy

**Affiliations:** 1 Department of Neurobiology, Care Sciences and Society, Division of Physiotherapy, 27106 Karolinska Institutet, Stockholm, Sweden; 2 Theme Womens Health and Allied Health Professionals, 27106 Karolinska University Hospital, Stockholm, Sweden; 3 Research and Development Unit, Stockholm Sjukhem Foundation, Stockholm, Sweden; 4 Department of Health Care Sciences, Marie Cederschiöld University, Stockholm, Sweden

**Keywords:** caregivers, digital health, Parkinson disease, qualitative research, self care

## Abstract

**Background:**

The rapid growth in eHealth has opened new possibilities for people with Parkinson’s Disease (PD). However, given the complexity of PD symptoms, the successful adoption of eHealth likely depends on support from family caregivers. While previous research has explored the perspectives of people with PD, insights into family caregivers’ perceptions of eHealth remain limited.

**Objective:**

To explore family caregivers’ perceptions of eHealth in formal care and in supporting physical activity and exercise.

**Methods:**

A qualitative design was used. Interviews were conducted with 15 family caregivers (mean age 72 years, 12 women) from rural and urban Sweden. Data were analyzed using inductive qualitative content analysis.

**Results:**

Three themes were formed. *Being the driving force for home, health and technology* described family caregivers’ responsibility for managing technology, balancing confidence with concerns about added burden and emotional strain, and supporting their partner’s engagement. *A threat to or enabler of quality time* reflected hopes that eHealth could save time and offer greater flexibility to schedule exercise and activities around daily routines, while also raising concerns about isolation and reduced social interaction if digital solutions replaced in-person contact. This theme also emphasized the need for accessible exercise solutions tailored to PD symptoms. *Bridging the gaps: blending digital and in-person care* highlighted reliance on online information due to limited professional guidance, appreciation for timely access to PD nurses, and a preference for integrated care models combining in-person care and digital follow-up.

**Conclusion:**

In conclusion, family caregivers highlighted both opportunities and challenges with eHealth in PD care, valuing its potential to ease daily responsibilities and enhance flexibility, while expressing concerns about added burden, reduced social interaction, and limited professional guidance.

## Introduction

Family caregivers are essential as providers of care and support to people with Parkinson’s Disease (PD).^1^ Currently, PD is the fastest-growing neurological disorder,^2^ projected to affect more than 11.8 million people globally by 2030.^3^ In consideration of the wider impact of the disease on daily life, including the lives of family caregivers, the proportion of people affected by PD is substantially higher.^1^ This multifaceted and progressive disease is characterized by both motor and non-motor symptoms, which eventually impair functional ability and quality of life.^4,5^ Research shows that support from partners is a significant motivator for initiating and maintaining a physically active lifestyle in this population.^6^ As of today, all available therapy options for PD are symptomatic, meaning that they do not affect the underlying neurodegenerative process.^5^ Engaging in routine symptom self-management, through physical activity and exercise, is therefore a vital complement to medical therapy. Physical activity and exercise has demonstrated benefits for both motor^7^ and non-motor symptoms^8^ and can enhance overall quality of life.^7^

Life expectancy after PD onset is highly variable. Survival typically varies between 7 to 14 years and is influenced by factors such as age of onset and presence of dementia.^9^ Given that life with PD can involve progressive decline spanning one or more decades, feelings of distress and burden are inevitable among family caregivers. These emotions tend to intensify as motor symptoms worsen and the person with PD becomes more reliant on assistance for daily activities.^10–12^ This is an ongoing challenge for family caregivers who, in addition to being the key provider of care and the coordinator of multiagency care, also have to manage their own health problems.^13^ A range of PD-related neuropsychiatric symptoms, such as depression, anxiety, and cognitive decline, can further exacerbate the burden on family caregivers.^14^ An added challenge is the change which incurs in interpersonal relationships. Family caregivers describe how the gradual shift in power dynamics creates a sense of losing the relationship they once used to have.^15^ Interestingly, the emotional aspects of care are described as more challenging than the physical care.^16^

The rapid development of digital technology and eHealth solutions over the past decades has created numerous opportunities for people with PD, their family caregivers, and healthcare professionals. Through mobile apps, wearable sensors, and web platforms, patients can monitor motor and non-motor symptoms, take part in exercise programs, have video consultations with healthcare professionals, and more. In theory, eHealth can improve communication between patients and care providers and may support more individualized care pathways, depending on technology design and implementation.^17^ The Swedish government articulated a “Vision for eHealth” in 2016, in which it envisioned that Sweden would become a world leader in the use of eHealth by 2025.^18^ Indeed, internet use is widespread in Sweden, with 97% of the population online. Moreover, four in ten people report that digital healthcare services make it easier to book a doctor’s appointment. However, one in ten instead feels that the shift toward eHealth has made access more difficult, a challenge that is particularly evident among older adults.^19^ Consistent with this, recent Swedish research shows that lower eHealth literacy is strongly linked to older age, particularly reflected in greater difficulty locating, understanding, and using online health information, navigating digital technologies, and accessing functional eHealth services.^20^

Given the complexity of PD symptoms, the adoption of eHealth is presumably reliant on support from the family caregiver.^1^ Although the perspectives of people with PD have been partly explored,^21,22^ our knowledge of PD family caregivers’ views and experiences with eHealth is limited. For research, and ultimately health care, to be able to effectively implement eHealth solutions in PD, it is therefore imperative that the voices of family caregivers be considered. The aim of this study was therefore to explore the perceptions of family caregivers to people with PD regarding the potential for eHealth in formal care contexts, as well as in supporting physical activity and exercise.

## Method

### Design, recruitment and participants

A qualitative approach with a descriptive and interpretative design was employed to explore the subjective experiences of family caregivers to individuals with PD. Family caregivers were recruited by advertisement in patient organization publications, via advertisement on social media platforms, and through contact with former research participants from the STEPs trial (registered at clinical trials, NCT05510739). A purposeful sampling strategy was used,^23^ in which recruitment focused on family caregivers who actively provided support to a person with PD which enabled physical activity, exercise or participation in social activities. This intention was clearly communicated in the advertisement and followed up during the initial telephone contact. Potential participants were contacted via telephone by the first author and informed about the purpose of the study prior to the interview being scheduled. In total, 15 family caregivers were included, whereof 14 were a married spouse and one was the sibling of an individual with PD. The sample size was guided by the concept of information power, meaning that studies with a focused aim, specific and relevant participants, theoretical grounding, and rich dialogue require fewer participants to achieve analytic depth.^24^ Given our study’s focused aim, and the high specificity of the sample, 15 interviews were considered sufficient to provide adequate information power. The family caregivers had a mean age of 72 years (ranging from 59 to 80 years), and the sample included 12 women and three men. Four of the participants were working part- or full-time, and 11 were retired. Eleven participants resided in an urban area, and four in a rural setting. Ten family caregivers reported that they experienced that the individual with PD they supported had cognitive decline, two of whom had an established dementia diagnosis. One family caregiver, and eight of the people with PD they supported used a mobility aid of some kind. All participants had some prior experience with eHealth, for example through the use of national e-services or digital appointment reminders, and a few also reported previous exposure to home-based digital exercise applications used by the person with PD whom they supported. The reporting in this study follows the COREQ (COnsolidated criteria for REporting Qualitative research) Checklist,^25^ see Suppementary Information.

### Ethical considerations

The study was approved by the Swedish Ethical Review Authority (2024-02339-01), and all participants signed a written informed consent.

### Data collection

Individual, in-depth, semi-structured interviews following an interview guide that included predefined themes and open-ended questions were conducted. Two theoretical frameworks; the Consolidated Framework for Implementation Research (CFIR)^26^ and the Theory of Self-Care of Chronic Illness,^27^ guided the development of the interview guide. Themes of questions were formulated around the following CFIR constructs: Knowledge and beliefs about the intervention, Self-efficacy, and Individual stage of change, and within the constructs questions were added relating to the three concepts from the Theory of Self-Care of Chronic Illness (self-care maintenance, self-care monitoring, and self-care management). The interview guide also contained opening questions, for example *“Can you start by telling me a little about who you are, and what your relationship is with [name]”* and *“What activities do you need to support your relative/partner in?”*, and a closing question *(“Is there anything else you would like to add about eHealth and physical activity that we haven’t talked about today?”*). The full interview guide is provided in Suppplementary Information. The first author (female), with previous experience in qualitative interviewing, conducted all interviews. The interviewer is a qualified physiotherapist but now works as a researcher. In an attempt to minimize response bias in relation to questions about physical activity and exercise, the interviewer presented herself as a researcher. Before each interview commenced, the interviewer read aloud a definition of eHealth, specifically the one used by the Swedish National Board of Health and Welfare and gave examples of digital tools/welfare technology to familiarize the interviewees with the concepts. The interviews were held either at Karolinska Institutet, in the participants’ homes, or digitally, based on participant preference. The interviews were recorded with a digital Dictaphone (Olympus VN-741PC) and transcribed word-for-word. Data collection occurred between October to November 2024, with interviews averaging 64 minutes in length (ranging from 34 to 85 minutes). Following each interview, field notes were systematically documented to capture contextual information, non-verbal expressions, and initial impressions relevant to the subsequent analysis.

### Data analysis

The data was analyzed using inductive qualitative content analysis according to the description by Graneheim and Lundman.^28,29^ Data management was conducted using NVivo software (Lumivero, version 15.2). After reading transcripts repeatedly, the first author identified and extracted meaning units, which comprised one or several sentences to ensure the preservation of contextual meaning.^28,29^ Coding was then performed by the first author whereby descriptive codes were applied to the meaning units and iteratively refined to accurately capture the nuances of the original text. The codes were kept detailed to support deeper contextual interpretation. The initial sorting of codes was conducted by the first author by organizing them into categories based on contextual similarities. The meaning units, developing codes, and preliminary category structures were then reviewed collaboratively with the co-authors. Although the co-authors did not review full transcripts, they were familiarized with the data through selected meaning units, and codes provided by the first author. These meetings enabled all authors to discuss the emerging categories, consider alternative interpretations, and refine the analytic structure. The sorting was initially validated by the last author (BL) and finalized in discussion with author AA. The interpretation of underlying meaning of each category was conducted in an iterative process involving discussions among all three authors. The categories were then combined into themes based on how they were interrelated. Data analysis continued until inductive thematic saturation was achieved, ensuring a comprehensive and nuanced representation of the emergent themes.^30^ Transcripts were not returned to participants for validation, and member checking was not conducted. Interviews and content analysis were conducted in Swedish, and the results and quotations were later translated into English. 

## Results

**Figure 1.**
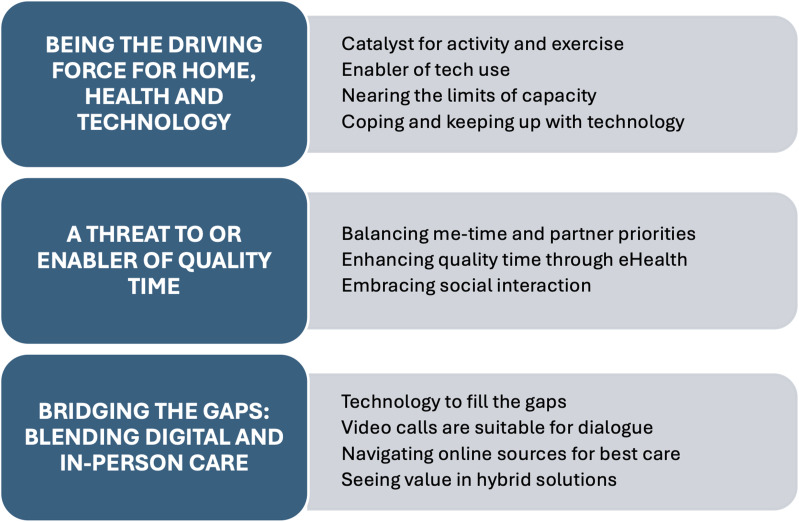
Overview of the three themes and their underlying categories.

The qualitative content analysis resulted in the formation of three themes: *Being the driving force for home, health and technology, A threat to or enabler of quality time,* and *Bridging the gaps: blending digital and in-person care*. An overview of the themes and their underlying categories is presented in Figure 1.

### Being the driving force for home, health and technology

The family caregivers often saw themselves as having the main responsibility for technology-related tasks and therefore felt they needed to stay current with digital tools and systems. While some family caregivers were optimistic and self-assured in their technology skills, others felt insecure as they perceived their skills as inadequate in the rapidly evolving digital landscape. They also noted that technological challenges were a source of frustration for the people with PD, potentially leading to decreased self-esteem. Family caregivers described already feeling overwhelmed by their current responsibilities and felt uncertain as to whether they would be able to handle even more. Specifically, several family caregivers were already shouldering the responsibility of housekeeping in addition to caring for their partner, and they feared that eHealth would further add to their load.

“I don't know, I feel like it's one more thing I have to take care of. I have to take care of his medicine, I have to take care of his hygiene, I have to take care of his... dressing and undressing. And then there's one more thing I have to do, there's like no time left for the fun things in life in a way.”

(Family caregiver 1, female)

Family caregivers described taking on a role of responsibility for initiating and motivating the person with PD to be active in everyday life, to an extent that they hadn’t before the diagnosis. This was not only taxing but could also give rise to a guilty conscience in moments when they themselves lacked the energy to support the partner’s activity.

”I try to push him. Because otherwise he will be sitting still a lot. Watching TV or sitting and reading or. He falls asleep as well so, it’s important to activate him as well”

(Family caregiver 13, female)

In addition to motivating and pushing the person with PD to be active, caregivers perceived needing to support their partner to a larger extent when technology was involved, when compared to before disease onset. Family caregivers whose partner had more advanced motor symptoms or cognitive decline often needed to assist them in using everyday technology, such as, mobile phones or the TV remote control. If exercise was to be performed in the home and it involved the use of technology, caregivers were often involved in preparing a suitable space and with starting up the program. Others described how symptoms of dopamine dysregulation syndrome required them to act as a gatekeeper for what happened online.

### eHealth - A threat to, or enabler of quality time

The family caregivers saw a potential for eHealth, especially in relation to remote consultations, which they perceived could free up time to engage in other more meaningful activities, for them and the person with PD. Travelling to and from healthcare appointments was not only time-consuming but could be difficult for those family caregivers who still worked. They also stressed that if exercise was to be conducted digitally, it was important that efforts were put in place to make it fun, easy to understand, and developed to work regardless of motor symptoms such as tremor.

“I feel like there are so many different appointments to go to now, so our time together is getting less and less, if you know what I mean. What happens to precious time?.…. we end up travelling around instead [to healthcare visits]. Of course, sometimes you have to, but sometimes I feel like, is this really worth it?”

(Family caregiver 13, female)

Some family caregivers were apprehensive that increased uptake of eHealth solutions would lead to increased isolation in the home, both for them and for the person with PD. They were particularly worried that the person with PD would lose valuable social interaction and were concerned about how this would negatively affect their well-being. Although the family caregivers acknowledged the importance of exercise to maintain and improve physical abilities for the person with PD, they considered group training sessions as equally important for the social element.

“That’s what I mean, that it triggers quite a lot because the social aspect was very important because it also makes you feel very good, the dopamine is probably created by this social aspect, not just by the training.”

(Family caregiver 5, female)

Something that featured repeatedly in the interviews was the experience among family caregivers of having less time for themselves. In order to care for their partner, they often sacrificed their own hobbies, meeting friends, or even their personal health-care needs. Family caregivers expressed a need to find time alone, or with friends, but recognized the need to put their partner first. While some described feeling almost bound to their partner, they also struggled with feelings of guilt when they left their partners to do things on their own.

“I tend to think or feel like I have two lives. I live two lives. I live my life and then I live [husband's name] life. Because I'm the one who lives his life, like very actively. And so it's always his life that comes first. That's how it is.”

(Family caregiver 14, female)

### Bridging the gaps: Blending digital and in-person care

The family caregivers reported that they often turned to online sources to find and verify information about symptoms, exercise advice, and medication regarding PD. This was largely due to a perceived lack of information provided by health care professionals. Although the family caregivers were expected to support their relative with PD and help monitor symptoms, guidance on how to do so was not always clearly communicated. In this context, contact with a PD nurse was described as especially valuable. The PD nurse was often easily reachable by telephone and could provide answers to questions or relay necessary information.

“If I'm going to be a support for the neurologist, I should have gotten some information, a few more facts about the disease. Because otherwise you don't know, as I once asked the neurologist, “how am I supposed to know what hyperkinesia is and what stiffness involves?”

(Family caregiver 3, male)

A shared perspective across the interviews was that participants believed that in-person meetings were essential for building a relationship between healthcare professionals and the person with PD. They therefore stressed the importance of having the first meeting in-person. They also believed that face-to-face interactions were more personal and facilitated easier questioning. Some participants believed that if the first meeting is clinic-based, then follow-up meetings could be done online. Participants reasoned that video calls specifically, could be suitable for healthcare professions who didn’t need to observe the entire body, but where dialogue was in focus. Family caregivers believed treatments with speech- and language pathologist and psychologists could be especially well suited to video consultation. Conversely, other professions like physical therapists or occupational therapists they believed were more dependent on physically being able to meet their patients or to see their home environment.

“I think I prefer personal meetings. At least with a physical therapist. It’s more hands-on if you are going to show movement, for touch and twist and turn or what not. But it will be a little more hands-on anyway. I could imagine a digital meeting with the neurologist. But a physical therapist, no, preferably in person.”

(Family caregiver 15, female)

Regardless of profession, family caregivers agreed that one important aspect, that was often lacking, was the occurrence of follow-up meetings. They shared examples where the person with PD was shown exercise routines by a physical therapist on one occasion, with no subsequent check-ins, or where mobility aids were fitted by an occupational therapist, who never followed up. This absence of continued support could result in exercises being neglected or mobility aids going unused. The family caregivers identified digital follow-ups as a promising solution to bridge this gap.

“But I think that if you had this, that you tied up the loose ends. That you kind of had a digital training. This is what you're going to do now. Three weeks ahead and once a day or so. And then you have this thing to do. It would be great if someone, on your side of things, could see how many times this has been done. Do you understand? And then you would actually have another conversation like this. And kind of ask how it went… I think you need that personal trainer part.”

(Family caregiver 7, female)

Although none of the family caregivers had experience with video consultations with a neurologist, they speculated that symptoms would be missed and that it would be difficult to avail fully of professional competence when assessment occurred from behind a screen. Similarly, they expressed concerns that the brief interactions during clinic-based healthcare visits did not reveal the full complexity of symptoms. They described how the person with PD often displayed a best version of themselves while at the neurologist office. It could also be that the family caregiver and the person with PD had conflicting views on the severity or impact of symptoms. Nonetheless, family caregivers, saw an opportunity to use digital technology as a means to prepare ahead of a health care visit. Filling out web surveys prior to a visit with the neurologist was, for example, described as a potential opportunity to raise awareness of symptoms and to discuss whether they and their partner perceived the effects of the disease in the same way. Others had considered the possibility of filming their partner to document the full extent of symptoms in everyday life, particularly during periods when the anti-Parkinson medication was wearing off, as these moments often revealed the most pronounced challenges.

“I've experienced that many times, that the right picture isn’t portrayed for the neurologist and I've said that, but it's a bit mean, but I've said that I'm going to film, can I film you so you can see how you walk? I think that could be useful for healthcare.”

(Family caregiver 5, female)

## Discussion

The findings indicate that family caregivers were ambivalent in their perceptions towards eHealth, both regarding healthcare visits, and as a means to support activity and exercise. They believed that increased use of technology would ultimately need to be handled by them, and some already described being at the brink of their capacity by taking care of the household as well as their partner. While they could see a potential for eHealth, they were also concerned that it might lead to further social isolation of their partner and further limit their own personal time. Family caregivers also believed that digital tools could be useful when preparing for and following up health care visits but emphasized the importance of initial clinic-based sessions to build trust.

As a person with PD experiences declining physical and cognitive abilities, domestic responsibilities gradually shift to the family caregiver, potentially causing negative impact on both individuals.^31^ In the current study, family caregivers described feeling overwhelmed with the responsibility of supporting their family member with PD in combination with taking care of the household. The support they provided wasn’t necessarily physical in nature but rather rooted in emotional and cognitive labor, involving continually initiating, planning, and motivating daily activities. Caregivers foresaw that increased use of eHealth or digital technology would ultimately have to be managed by them. This, they feared, would decrease their “me-time” even more. Previous research has documented that family caregivers of individuals with PD frequently report experiencing both social and emotional isolation, alongside an expressed need for personal time or respite.^32^ Finding time to spend with friends or doing leisure activities, so-called social self-management, can be an important strategy to decrease emotional burden for family caregivers.^33^ However, the family caregivers in this study also saw the time-saving potential of eHealth, a positive aspect also highlighted in earlier research in dementia.^34^

There is a strong need for information among family caregivers of people with PD,^35^ and some educational and psychoeducational programs have been developed to address this demand.^36,37^ Family caregivers in the current study, however, described not having received the information and support they felt they needed from healthcare to assist their family member with PD. They were, for example, expected to understand PD symptoms and manage medication, yet the information available was limited and primarily drawn from informal sources rather than structured healthcare guidance. This lack of access to information can serve to further increase caregiver burden.^11^ Previous research has also shown that when family caregivers experience a lack of entitlement to support from health care personnel, and when the primary focus is on the person with PD, they find it difficult to call out for emotional support for themselves.^38^ Our findings, and those of others, further highlight the disparities that can arise between how a family caregiver and the individual with PD perceive the severity of symptoms and ways of dealing with them.^39^ Interestingly, family caregivers in this study, viewed digital technology as a means to foster discussion on their disagreements and to provide a more comprehensive picture of their partners situation at the healthcare visit.

In the context of the current study, it is essential to recognize that each family caregiving situation is unique. According to the caregiver identity theory, the role as a family caregiver emerges from the existing relationship and evolves along the disease continuum.^40^ Furthermore, caregiving is governed by the ethos developed within each unique family, by ethnic and cultural backgrounds, and by norms and social roles.^40^ Over the past decade, various integrated care models for PD incorporating technological components, such as, online support and self-monitoring have been developed.^41,42^ The extent of co-design with family caregivers has however varied. Including caregivers in co-creation processes may help mitigate some of the expected burdens of eHealth by ensuring that digital tools and workflows align with their capabilities and everyday realities, as highlighted in a recent realist review of co-creation in digital health services for older adults.^43^ The findings of the current study emphasize how care models which integrate digitally supported self-care, will ultimately depend on family caregiver involvement for successful implementation. Considering the vital role of family caregivers in integrated and person-centred medicine and rehabilitation, future research should investigate ways to develop support systems, tailored to their individual needs.

The strengths of this study include a transparent and rigorous analysis process which resulted in varied descriptions of both positive and negative experiences and perceptions of eHealth. The family caregivers interviewed for this study resided in various parts of the country, including both urban and rural areas. Although all were able to speak and understand Swedish, not all had it as their first language, something which is highly reflective of the Swedish society. One limitation however is that all family caregivers but one was a spouse. Spouses often share a household and provide continuous support, which may result in caregiving experiences that differ from those of adult children, siblings, or other relatives who provide care from a distance or with different responsibilities. Therefore, the findings may not fully capture the diversity of caregiving perspectives across non-spousal relationship types. Furthermore, the sample consisted predominantly of older women, a demographic that may have specific patterns of technology use and varying digital literacy levels,^20^ which could have shaped their expectations and experiences of eHealth. The analysis may also be influenced by potential single-coder bias, as the initial coding was carried out by one researcher. This was addressed through collaborative discussions in which co-authors reviewed selected meaning units, codes, and the developing category structure. Finally, the absence of transcript validation and member checking represents a limitation, as participants did not review or comment on the interpretations.

In conclusion, family caregivers expressed ambivalence toward eHealth, recognizing its potential to assist daily life through time-saving and improved documentation, while also fearing it could increase their personal burden and further isolate the person with PD. These findings highlight an urgent need for research focused on how to effectively support family caregivers in their complex and emotionally demanding roles. It is essential that the development and implementation of eHealth interventions actively incorporate family caregivers’ lived experiences to ensure such technologies alleviate, rather than exacerbate, their burden.

## Supplemental material

Supplemental material - Balancing benefits and challenges of eHealth for family caregivers of people with Parkinson’s disease – A qualitative study Supplemental material for Balancing benefits and challenges of eHealth for family caregivers of people with Parkinson’s disease – A qualitative study by Hanna Johansson, Anette Alvariza and Breiffni Leavy in Digital Health.

## Data Availability

Due to Swedish and EU personal data legislation datasets generated during current study will not be made publicly available, but availability may be permitted by the corresponding author on reasonable request. Any sharing of data will be regulated via a data transfer and user agreement with the recipient.*
