# Supplementary material for: Balancing benefits and challenges of eHealth for family caregivers of people with Parkinson’s disease – A qualitative study
Source: Digit Health. 2026 Apr 29;12:20552076261447410. doi: 10.1177/20552076261447410 (PMC13150097; doi:10.1177/20552076261447410)
Supplement: Supplemental material - Balancing benefits and challenges of eHealth for family caregivers of people with Parkinson’s disease – A qualitative study [file sj-pdf-1-dhj-10.1177_20552076261447410.pdf]

Supplementary Information

Table of Contents

INTERVIEW GUIDE **2**

COREQ (CONSOLIDATED CRITERIA FOR REPORTING QUALITATIVE RESEARCH) CHECKLIST **4**

DOMAIN 1: RESEARCH TEAM AND REflEXIVITY 4
DOMAIN 2: STUDY DESIGN 4
DOMAIN 3: ANALYSIS AND fiNDINGS 6

CODING TREE **7**

THEME - BEING THE DRIVING FORCE FOR HOME, HEALTH AND TECHNOLOGY 7
Category – Catalyst for activity and exercise *7*
Category – Enabler of tech use *7*
Category – Nearing the limits of capacity *7*
Category – Coping and keeping up with technology *7*
THEME - A THREAT TO OR ENABLER OF QUALITY TIME 7
Category – Balancing me-time and partner priorities *7*
Category – Enhancing quality time through eHealth *8*
Category – Embracing social interaction *8*
THEME – BRIDGING THE GAPS: BLENDING DIGITAL AND IN-PERSON CARE 8
Category – Technology to fill the gaps *8*
Category – Video calls are suitable for dialogue *8*
Category – Navigating online sources for best care *8*
Category – Seeing value in hybrid solutions *9*

Interview guide

Opening questions

- Can you start by telling us a little about who you are, and what your relationship is with [name].

- How active is your relative/partner today?

  o What activities do you do together?

  o What activities do you need to support your relative/partner in?

- How physically and socially active was your relative/partner before?

  o How important were these different activities to you in your life? (if partner)

- How prioritized/important do you think it is that your relative receives support for physical activity or physical exercise?

- What type of support do you receive, or have you received previously, to be able to care for/take care of your relative/partner?

- What type of support do you feel you need to be able to care for/take care of your relative?

- What experiences do you have of using digital tools/welfare technology?

Knowledge & confidence in the intervention

- Do you think that eHealth/digital tools can be effective in helping you support your relative with physical activity? Why/why not? In what way? *(self-care maintenance)*

- Do you think that eHealth/digital tools can be effective in helping you support your relative in social activities? Why/why not? In what way?

- What are your thoughts about using eHealth as a tool for physical activity and/or exercise in your home and surrounding area?

- In what way do you think that eHealth could be used to, for example, keep track of symptoms and changes related to Parkinson's disease? *(self-care monitoring)*

  o Physical activity/steps per day

  o Falls

  o Freezing

  o Fluctuations

  o Medication

  o Other, e.g. being able to do tests at home?

- In what way do you think eHealth could be used in rehabilitation or other contact with healthcare? *(self-care management)*

- How do you see eHealth as a support for planning in everyday life?

- What content do you think would be good/valuable to get with a digital tool

  o Information about PS, training or other?

- How do you see being able to use digital tools to set goals?

  o Is goal setting something that healthcare professionals usually discuss with you or your relative? If so, in what way?

  o Do you have any suggestions on how goals should be set and how to involve relatives in this process?

Self-efficacy

- How confident/safe do you feel in using eHealth/digital tools?

- How confident/safe do you think your relative feels in using eHealth/digital tools?

Individual stage of change

- How prepared do you feel to use eHealth/digital tools?

- What things do you think could make it easier for you to use digital tools?

- What benefits do you see with eHealth/digital tools as support for physical activity/training?

- What challenges/obstacles do you see with eHealth/digital tools as support for physical activity/training?

Closing question

- Is there anything else you would like to add about eHealth and physical activity that we haven't talked about today? Other areas of use for digital tools that could make it easier for you as a relative?

COREQ (COnsolidated criteria for REporting Qualitative research) Checklist

Developed from:

Tong A, Sainsbury P, Craig J. Consolidated criteria for reporting qualitative research (COREQ): a 32-item checklist for interviews and focus groups. *International Journal for Quality in Health Care*. 2007. Volume 19, Number 6: pp. 349 – 357

**Domain 1: Research team and reflexivity**		
**Topic**	**Guide questions/description**	**Reported on Page No.**
Personal Characteristics		
Interviewer/facilitator	Which author/s conducted the interview or focus group?	8
Credentials	What were the researcher's credentials? E.g. PhD, MD	8
Occupation	What was their occupation at the time of the study?	8
Gender	Was the researcher male or female?	8
Experience and training	What experience or training did the researcher have?	8
Relationship with participants		
Relationship established	Was a relationship established prior to study commencement?	6
Participant knowledge of the interviewer	What did the participants know about the researcher? e.g. personal goals, reasons for doing the research	6
Interviewer characteristics	What characteristics were reported about the interviewer/facilitator? e.g. Bias, assumptions, reasons and interests in the research topic	6

**Domain 2: study design**		
**Topic**	**Guide questions/description**	**Reported on Page No.**

Theoretical framework		
Methodological orientation and Theory	What methodological orientation was stated to underpin the study? e.g. grounded theory, discourse analysis, ethnography, phenomenology, content analysis	8
Participant selection		
Sampling	How were participants selected? e.g. purposive, convenience, consecutive, snowball	6
Method of approach	How were participants approached? e.g. face-to-face, telephone, mail, email	6
Sample size	How many participants were in the study?	6
Non-participation	How many people refused to participate or dropped out? Reasons?	6
Setting		
Setting of data collection	Where was the data collected? e.g. home, clinic, workplace	8
Presence of non-participants	Was anyone else present besides the participants and researchers?	8
Description of sample	What are the important characteristics of the sample? e.g. demographic data, date	6-7
Data collection		
Interview guide	Were questions, prompts, guides provided by the authors? Was it pilot tested?	7-8
Repeat interviews	Were repeat interviews carried out? If yes, how many?	No
Audio/visual recording	Did the research use audio or visual recording to collect the data?	8
Field notes	Were field notes made during and/or after the interview or focus group?	8
Duration	What was the duration of the interviews or focus group?	8
Data saturation	Was data saturation discussed?	9
Transcripts returned	Were transcripts returned to participants for comment and/or correction?	No, see page 9

Domain 3: analysis and findings		
**Topic**	**Guide questions/description**	**Reported on Page No.**
Data analysis		
Number of data coders	How many data coders coded the data?	8-9
Description of the coding tree	Did authors provide a description of the coding tree?	Yes, see Supplementary Information
Derivation of themes	Were themes identified in advance or derived from the data?	8-9
Software	What software, if applicable, was used to manage the data?	8
Participant checking	Did participants provide feedback on the findings?	No, see page 9
Reporting		
Quotations presented	Were participant quotations presented to illustrate the themes/findings? Was each quotation identified? e.g. participant number	Yes, see page 10-15
Data and findings consistent	Was there consistency between the data presented and the findings?	10-15
Clarity of major themes	Were major themes clearly presented in the findings?	10-15
Clarity of minor themes	Is there a description of diverse cases or discussion of minor themes?	16-19

CODING TREE

Theme - Being the driving force for home, health and technology

Category – Catalyst for activity and exercise

Codes:
- Having to push to get activity done
- Bad conscience
- Feeling frustrated
- Lack of initiative and motivation
- More effective if training is on site
- Remind home care about activity
- Support to start training

Category – Enabler of tech use

Codes:
- Adapting home for training
- Having to attend is a barrier
- Need help getting started
- Being the gatekeeper for what happens online
- Cognitive impairments are limiting
- Motor symptoms are limiting
- Worried about the future

Category – Nearing the limits of capacity

Codes:
- Responsibility increases with eHealth
- At a tipping point
- Being the main link to healthcare
- I need physical help, not digital
- I am a hobby neurologist
- Digital call support
- Safety during home training

Category – Coping and keeping up with technology

Codes:
- Trying to stay up to date
- Feeling confident with technology
- Being forced into digital development
- A generational problem
- Digital technology can cause stress
- Frustration when technology is complicated
- Can cause lower self-esteem in partner
- Partner unfamiliar with digital technology

Theme - A threat to or enabler of quality time

Category – Balancing me-time and partner priorities

Codes:
- Feeling tied up
- Needs a breather

Needs to do things himself
His life comes first
Unbearable if you don't do anything

Category – Enhancing quality time through eHealth

Codes: Making it fun
Needs to be easy
Digital training an option in the future
Increased flexibility with your exercise
Maybe I'm doing too much
The screen needs to be big
No need to travel to healthcare
No need to sit in a phone queue
Saves time
Tech can enable autonomy
Our time is valuable

Category – Embracing social interaction

Codes: Partner needs to get out of the house
Being social with digital tools
The importance of physical meetings
The importance of social interaction

Theme – Bridging the gaps: blending digital and in-person care

Category – Technology to fill the gaps

Codes: Better than nothing
Coming prepared
Can increase motivation
Cognitive training
Monitor tremor
Structure and plan
Visualize progression
Assessment becomes more difficult digitally
Competence lost behind the computer

Category – Video calls are suitable for dialogue

Codes: Digital meetings can be suitable for dialogue
Digital meetings with speech therapist
Digital support from a psychologist
Advantage of being able to see the person you are talking to
Video meeting quite similar to a physical meeting

Category – Navigating online sources for best care

Codes: Responsibility for finding and conveying information

Incorrect information if not included in visit
Information requests

Category – Seeing value in hybrid solutions

Codes: Asking questions face-to-face is superior
Face-to-face is always better
Face-to-face to establish contact
Physical meetings are more personal
You don't have to see each other all the time
Conversation quality is better in-person
Follow-ups digitally
It's important to have physical visits too
